# Convergent losses of arbuscular mycorrhizal symbiosis in carnivorous plants

**DOI:** 10.1111/nph.70544

**Published:** 2025-09-25

**Authors:** Héctor Montero, Matthias Freund, Kenji Fukushima

**Affiliations:** ^1^ Center for Frontier Research National Institute of Genetics Mishima 411‐4540 Japan; ^2^ Institute for Molecular Plant Physiology and Biophysics University of Würzburg Würzburg 97082 Germany; ^3^ Genetics Program Graduate Institute for Advanced Studies, SOKENDAI Mishima 411‐4540 Japan

**Keywords:** arbuscular mycorrhizal symbiosis, carnivory, convergent evolution, gene loss, nutritional strategy

## Abstract

- Most land plants form the ancient arbuscular mycorrhizal (AM) symbiosis, while carnivory is a younger trait that evolved in several angiosperm orders. The two biotic interactions similarly help plants acquire mineral nutrients, raising the question of whether they can coexist. However, the mycorrhizal status of carnivorous plants has long remained speculative.
- We surveyed the occurrence of AM‐associated genes across carnivorous plant lineages, performed AM fungal inoculation assays, and microscopically evaluated the patterns of colonization.
- We found convergent losses of the AM trait either coincident with or predating the emergence of carnivory. Exceptionally, the carnivorous plant *Roridula gorgonias* retains symbiosis‐related genes and forms arbuscules. The youngest carnivorous lineage, *Brocchinia reducta*, showed signatures of the early stages of AM trait loss. An AM‐associated *CHITINASE* gene encodes a digestive enzyme in the carnivorous plant *Cephalotus*, suggesting gene co‐option.
- We uncovered a mutually exclusive trend of AM symbiosis and carnivory, with only rare instances of coexistence. These findings illuminate the largely unexplored processes by which plant nutritional strategies evolve and supplant one another over time.

## Resumen

- La mayoría de las plantas terrestres forman la ancestral simbiosis micorrízica arbuscular (MA), mientras que la carnivoría es un rasgo más reciente que evolucionó en varios órdenes de angiospermas. Ambas interacciones bióticas ayudan a las plantas a adquirir nutrientes minerales, lo que plantea la cuestión de si pueden coexistir. Sin embargo, el estado micorrízico de las plantas carnívoras ha sido objeto de especulación durante mucho tiempo.
- Estudiamos la presencia de genes asociados a la MA en linajes de plantas carnívoras, realizamos ensayos de inoculación de hongos MA y evaluamos microscópicamente los patrones de colonización.
- Encontramos pérdidas convergentes del rasgo MA coincidentes o anteriores a la aparición de la carnivoría. Excepcionalmente, la planta carnívora *Roridula gorgonias* conserva genes relacionados con la simbiosis y forma arbúsculos. El linaje carnívoro más reciente, *Brocchinia reducta*, mostró indicios de las primeras etapas de la pérdida del rasgo MA. Un gen CHITINASE asociado a AM codifica una enzima digestiva en la planta carnívora Cephalotus, lo que sugiere una cooptación génica.
- Descubrimos una tendencia mutuamente excluyente entre la simbiosis MA y la carnivoría, con solo raros casos de coexistencia. Estos hallazgos arrojan luz sobre los procesos, en gran parte inexplorados, mediante los cuales las estrategias nutricionales de las plantas evolucionan y se sustituyen mutuamente con el tiempo.

## Introduction

Nutrient acquisition is pivotal for plant fitness, and evolution is expected to select for traits that efficiently modulate this process. Plants are able to capture mineral nutrients directly from their environment and by interacting with other organisms.

Most land plants form a mutualistic relationship known as the arbuscular mycorrhizal (AM) symbiosis with fungi from the subphylum Glomeromycotina. This is an ancient interaction that is believed to have enabled plant transitions from aquatic to terrestrial habitats and that accompanied early land plants, predating in millions of years the evolution of roots or flowers (Strullu‐Derrien *et al*., 2018; Rich *et al*., 2021). In return for photosynthesis‐fixed carbon, AM fungi provide plants with mineral nutrients such as phosphorus, nitrogen and potassium (Lanfranco *et al*., 2018).

During the course of land plant evolution, several lineages have independently lost the ability to establish symbioses with AM fungi. This scenario is usually accompanied by plants partnering with other root symbionts or by evolving alternative nutrient acquisition strategies including parasitism and the formation of specialized roots adapted for phosphate scavenging such as those in the families Proteaceae and Cyperaceae. The non‐AM trait is also found among plants that lack developed underground roots including some epiphytes and aquatic species (Lambers & Teste, 2013). Cases also exist where AM symbiosis is absent in plants with seemingly ordinary lifestyles, notably in the Brassicaceae (Sharma *et al*., 2023). Phylogenomic approaches have been used to identify AM symbiosis‐associated genes (hereafter symbiosis genes) that are lost upon mutualism breakdown (Delaux *et al*., 2014; Favre *et al*., 2014; Bravo *et al*., 2016). Exploring the evolutionary paths leading to the loss of the AM symbiosis would be beneficial for better understanding how plants optimize their nutrient acquisition strategies.

Plant carnivory is a convergently evolved trait that is evolutionarily younger than AM symbiosis and has arisen in six angiosperm orders (Fleischmann *et al*., 2018; Lin *et al*., 2021). Carnivory takes place mainly by means of highly specialized leaves that form traps, capable of capturing, killing, and digesting arthropod prey. The nutrients acquired from prey are of similar nature to those that plants acquire from AM fungi. This posits questions on functional redundancy between the two nutritional traits, AM symbiosis and carnivory. However, research on biotic interactions in carnivorous plants usually centers more on prey than on root biology (Montero & Fukushima, 2023), and whether or not carnivorous plants can form mycorrhizae is uncertain. Past studies (see below) aimed to analyze their root microbiota in natural communities with equivocal outcomes, and no study has attempted inoculation assays. One study reported the presence of AM fungal DNAs in an attempt to analyze the root endophytes in the flypaper‐type carnivorous plant *Drosera rotundifolia* (Quilliam & Jones, 2010). Another study made microscopic examinations in this species and found aseptate hyphae and vesicles, but no arbuscules (Weishampel & Bedford, 2006). Reports also exist on low colonization and presence of arbuscules in *Drosera intermedia*, but no microscopy image is provided (Fuchs & Haselwandter, 2004). In another publication, arbuscules are said to occur in *Drosera burmannii* and *Drosera indica* (Harikumar, 2013), but the images provided do not show arbuscules and may not correspond to AM fungi. Coincidently, all these studies had made *Drosera* their study subject, a group of carnivorous plants in the Caryophyllales. Dedicated studies in other carnivorous plants are lacking. Recent research compiles these incomplete observations into a large‐scale dataset to derive evolutionary insights (Werner *et al*., 2018). Altogether, the mycorrhizal status of carnivorous lineages lacks unequivocal evidence, with the unsubstantiated notion of them being non‐mycorrhizal (Adlassnig *et al*., 2005).

The convergent evolution of carnivory provides an excellent opportunity to test the patterns of AM symbiosis loss associated with the emergence of alternative nutritional strategies. In order to scrutinize possible connections between the independent losses of AM symbiosis and the independent gains of plant carnivory, we undertook a comparative genomics approach to explore the presence of symbiosis‐related genes in carnivorous plants across angiosperms. Additionally, we coupled this with inoculation assays and microscopic examinations. Our analyses suggest that AM symbiosis has been independently lost in most, but not all, carnivorous lineages.

## Materials and Methods

### Species set

We surveyed for coding DNA sequence (CDS) Fasta files among publicly available genomes in the target orders Poales, Oxalidales, Caryophyllales, Ericales, and Lamiales, totaling 82 genomes. One species per genus was included, except in carnivorous plants, where all available were used. In the case of the Poaceae, which has many genomes available, one genome per subfamily was included. Likewise, we surveyed and collected transcriptomes in all families harboring carnivorous plant species and also in species belonging to families not represented in the genome dataset within the five target orders, totaling 90 transcriptomes. We supplemented this with newly generated genomes and transcriptomes from a total of 15 species, many of them being carnivorous. These data were generated as part of a separate study, but transcriptome assemblies and genome‐based coding sequence sets needed to reproduce this study are fully available in the supplementary data (see the Data availability section). Additionally, we included 40 genomes outside of the focal orders, aiming for the representation of all angiosperm orders with genomes available. This resulted in a final dataset of 123 genomes and 103 transcriptomes. File sources are listed in Supporting Information Table S1.

### Transcriptome assembly and contamination removal

Publicly available RNA‐seq data were retrieved and preprocessed using Amalgkit v.0.9.34 (https://github.com/kfuku52/amalgkit), which internally uses Parallel‐Fastq‐dump v.0.6.7 (https://github.com/rvalieris/parallel‐fastq‐dump) and Fastp v.0.23.2 (https://github.com/OpenGene/fastp). Transcriptome assembly was performed using up to 30 Gb of RNA‐seq reads per species with Trinity v.2.13.2 (https://github.com/trinityrnaseq/trinityrnaseq). Open reading frames (ORFs) were identified using TransDecoder v.5.5.0, with a minimum length threshold of 50 bp (‐m 50) (https://github.com/TransDecoder/TransDecoder). The longest ORFs among isoforms were selected using the ‘aggregate’ function in Cdskit v.0.10.9 (https://github.com/kfuku52/cdskit). To prevent false detection of potentially contaminated sequences, taxonomic assignment was conducted using MMseqs2 v.13.45111 (https://github.com/soedinglab/MMseqs2), and sequences assigned to inconsistent phyla were removed. Finally, the remaining sequences were compiled to generate representative CDSs. Translated CDSs were analyzed using Rps‐Blast v.2.13.0 against Pfam‐A families (released on November 25, 2020) with an *E*‐value cutoff of 0.01 to determine protein domain architectures.

### Species tree inference

The species tree was inferred as described previously (Saul *et al*., 2023), utilizing a total of 1614 single‐copy genes identified by benchmarking universal single‐copy orthologues (Busco) v.5.3.2 (https://gitlab.com/ezlab/busco) with the Embryophyta dataset in OrthoDB v.10 (embryophyta_odb10). In‐frame codon alignments were generated by first aligning translated protein sequences using Mafft v.7.520, followed by trimming and back‐translation with TrimAl v.1.4.1 (https://github.com/inab/trimal) to obtain in‐frame codon alignments. For each single‐copy gene, nucleotide and protein maximum‐likelihood (ML) trees were constructed using IQ‐Tree v.2.2.2.7 (https://github.com/iqtree/iqtree2) with the GTR + R4 and LG + R4 models, respectively. The collection of 1614 single‐copy gene trees was then subjected to coalescence‐based species tree inference using Astral v.5.7.3 (https://github.com/smirarab/ASTRAL). Additionally, concatenated alignments were used as input for nucleotide‐ and protein‐based ML tree inference with the aforementioned substitution models. *Amborella trichopoda* Baill. was used as the outgroup for rooting. The protein‐based ML tree was employed as the species tree for subsequent phylogenetic reconciliation analyses.

### Symbiosis gene set

Symbiosis genes used here were largely identified in a previous phylogenomics study (Bravo *et al*., 2016). Some genes were included as they were shown to have an AM‐associated pattern in later studies, including *CCaMK*, *LIN/LINL* (Radhakrishnan *et al*., 2020), and *LysMe* (Yu *et al*., 2023). In total, this corresponds to 75 genes (Table S2). Symbiosis genes were identified in our dataset by searching for the orthogroups containing the *Medicago truncatula* Gaertn. orthologs, whose IDs are provided in Bravo *et al*. (2016). Gene IDs of other model species were either obtained from the relevant literature when the genes were characterized or, when not characterized, from the Ortholog Search function in the Tomato Symbiotic Transcriptome Database (https://efg.nju.edu.cn/TSTD/; Zeng *et al*., 2023).

### Orthogroup tree inference

To ensure robust conclusions, we used two different approaches for orthogroup classification: a phylogenetic approach and a graph‐based approach. For phylogenetic orthogroup extraction, a tBlastx search (v.2.14.0; Camacho *et al*., 2009) was first performed against the 229 CDS sets using preselected symbiosis genes as queries, with an *E*‐value cutoff of 0.01 and a minimum Blast hit coverage of 25% on the query sequence, to obtain up to 5000 homologous sequences. Next, an ML phylogenetic tree was inferred using IQ‐Tree with the GTR + G4 nucleotide substitution model, following the generation of in‐frame codon sequence alignments as described above. Trees containing > 2000 sequences were inferred with the ‘‐‐fast’ option. After species‐tree‐guided gene tree rooting (Fukushima & Pollock, 2020), which internally utilizes Notung v.2.9 (https://www.cs.cmu.edu/~durand/Notung/), the phylogenetic orthogroups containing the Blast query sequences were finally extracted using Nwkit v.0.11.10 (https://github.com/kfuku52/nwkit) with the ‘‐‐orthogroup’ option. The graph‐based orthogroup inference was conducted using SonicParanoid v.2.0.5a2 (https://gitlab.com/salvo981/sonicparanoid2) with representative CDS sets from 229 species, resulting in a total of 110 791 orthogroups from which symbiosis gene orthogroups were identified. ML trees were inferred using the same procedure. All orthogroup trees obtained from the two methods were subjected to the evaluation of tree topology confidence using ultrafast bootstrapping (‐‐ufboot 1000) and optimized through hill‐climbing nearest neighbor interchange (‐bnni) in IQ‐Tree. The ML tree was then used as the starting gene tree for GeneRax v.2.0.4 (https://github.com/BenoitMorel/GeneRax) to generate a rooted, species‐tree‐aware orthogroup tree.

### Symbiosis gene occurrence

Using the SonicParanoid and Blast‐based trees, the occurrence of the different symbiosis genes was assessed in the species within the dataset. In the case of SonicParanoid trees, different symbiosis genes were occasionally encountered in a common orthogroup. The situation where one symbiosis gene was split into two orthogroups also occurred. Blast‐based trees were also commonly composed of the target symbiosis gene and their close nonsymbiosis paralogues. In these cases, symbiosis genes were discerned by searching the known *M. truncatula* and rice symbiosis gene orthologs. The subtrees containing them, having an *A. trichopoda* as the basalmost ortholog, were individualized, and all the genes contained in these subtrees were considered part of the symbiosis gene group, irrespective of lineage‐specific duplications, genes coding for seemingly incomplete proteins, or genes with misplaced phylogenetic positions. Symbiosis genes were considered present if they were present in either the SonicParanoid tree or their Blast‐based tree counterparts. Trees can be found in the supplementary data (see the Data availability section).

### Genome‐wide analysis of convergent gene losses

To study genes commonly lost in carnivorous plants, we determined orthogroups that were absent in all analyzed carnivorous plant genomes and transcriptomes in the Oxalidales and Lamiales, specifically, *Cephalotus follicularis* Labill. (Oxalidales), and all species in the genera *Byblis*, *Genlisea*, *Pinguicula*, and *Utricularia* (Lamiales) and present in the noncarnivorous genomes of *Averrhoa carambola* L. (Oxalidales), *Andrographis paniculata* (Burm.f.) Wall. ex Nees, and *Erythranthe guttata* (DC.) G.L.Nesom (Lamiales). Table S3 includes all data required to reproduce this analysis or to explore alternative species sets.

### Arbuscular mycorrhizal inoculation assays

Young *Brocchinia reducta* Baker, *Brocchinia acuminata* L.B.Sm., *Roridula gorgonias* Planch., and *Stylidium debile* F.Muell. plants were obtained from a commercial carnivorous plant nursery (Gartenbau Thomas Carow, Nüdlingen, Germany). *Cephalotus follicularis* plants were maintained in tissue cultures (Fukushima *et al*., 2017). At least three plants of a given species were analyzed per experiment, with the exception of *B. acuminata*. In this case, we procured a single individual. Roots were carefully washed before transferring plants to pots with autoclaved sand as a substrate. AM fungal inoculum was applied. One thousand spores per pot of the AM fungal species *Rhizophagus irregularis* (Blaszk., Wubet, Renker & Buscot) C. Walker & A. Schüßler (Agronutrition, Carbonne, France) were used as inoculum. In the case of *Funneliformis mosseae* (T.H. Nicolson & Gerd.) C. Walker & A. Schüßler, 1 ml of crude inoculum (MycAgro Lab, Breteniere, France) was applied. Plants were grown in the glasshouse for 6 wk under a low phosphate fertilization regime administered once a week in the form of 10 μM KH_2_PO_4_.

### Arbuscular mycorrhizal fungal staining and microscopy

At six wpi, roots were harvested and stained with Trypan blue, largely taking place as described previously (Montero *et al*., 2021), with an exception for *R. gorgonias* dark roots, where the clearing step consisted of 20% (w/v) potassium hydroxide (KOH) replaced every other day in a span of a week. Roots were mounted in glass microscope slides, observed in an Olympus CX21 microscope (Olympus, Hamburg, Germany), and images were acquired using an affixed FUJIFILM X‐T2 camera (FUJIFILM, Ratingen, Germany). For confocal microscopy, roots were stained with Wheat Germ Agglutinin (WGA), CF®488A Conjugate (Biotum, Fremont, CA, USA). Root sections were excised and incubated in 50% (v/v) ethanol for 1 h, followed by 20% (w/v) KOH for 2 d, and 0.1 M HCl for 2 h. A 0.2 μg ml^−1^ WGA‐CF®488A solution in 1× phosphate‐buffered saline (PBS, pH 7.4) was added, and samples were incubated at 4°C in the dark for 1 wk. Imaging was carried out in a confocal laser scanning microscope (Leica TCS SP5 II; Leica Microsystems, Wetzlar, Germany). WGA‐CF®488A was detected with an excitation wavelength of 488 nm, and emitted wavelengths were collected at 492–533 nm. Roots were observed using a 40× water immersion objective. Image processing took place using Fiji/ImageJ (Schindelin *et al*., 2012).

## Results

### Occurrence of symbiosis genes in carnivorous plants

In order to determine the occurrence of symbiosis‐related genes in carnivorous lineages, we generated a dataset of 124 genomes and 105 transcriptomes centered on five plant orders where carnivory evolved and representatives in the rest of angiosperm orders. Publicly available data were supplemented by a set of newly generated transcriptomes from multiple tissues, including roots. The symbiosis genes we used were previously identified through a phylogenomic method (Bravo *et al*., 2016). In this original publication, symbiosis genes were identified using a cohort of *c*. 50 plant species. They determined which genes were present in AM hosts and which were absent in known nonhost species such as Arabidopsis, sacred lotus, and duckweed. Many of these genes had either a known function in the AM symbiosis at the time of publication or were found to be involved in the association since then, illustrating the reliability of the gene list. We identified the orthologs of these 75 symbiosis genes across the genomes in our dataset. First, we employed two distinct approaches for orthogroup assignment: a graph‐based tool, SonicParanoid2 (Cosentino *et al*., 2024), and a Blast sequence search combined with phylogenetic orthogroup identification (see the Materials and Methods section). Next, we reconstructed a species‐tree‐aware ML gene tree for each orthogroup to accurately detect orthologous relationships, even in cases of complex evolutionary histories involving lineage‐specific gene duplications and losses. Finally, we reported the occurrence of symbiosis genes if one or more orthologs were detected by either or both orthogroup construction methods. Genome sequencing, gene prediction, and transcriptome assembly do not guarantee the complete recovery of gene sets, making it difficult to distinguish between genuine gene loss and technical omissions for individual genes. However, gene set completeness scores were high for genomes (Busco completeness = 89.5 ± 8.6%, mean ± SD, *n* = 124 species) and transcriptomes (77.1 ± 18.1%, *n* = 105), indicating that our dataset provides sufficient gene coverage to capture gene retention trends across multiple species and genes.

**Fig. 1:**
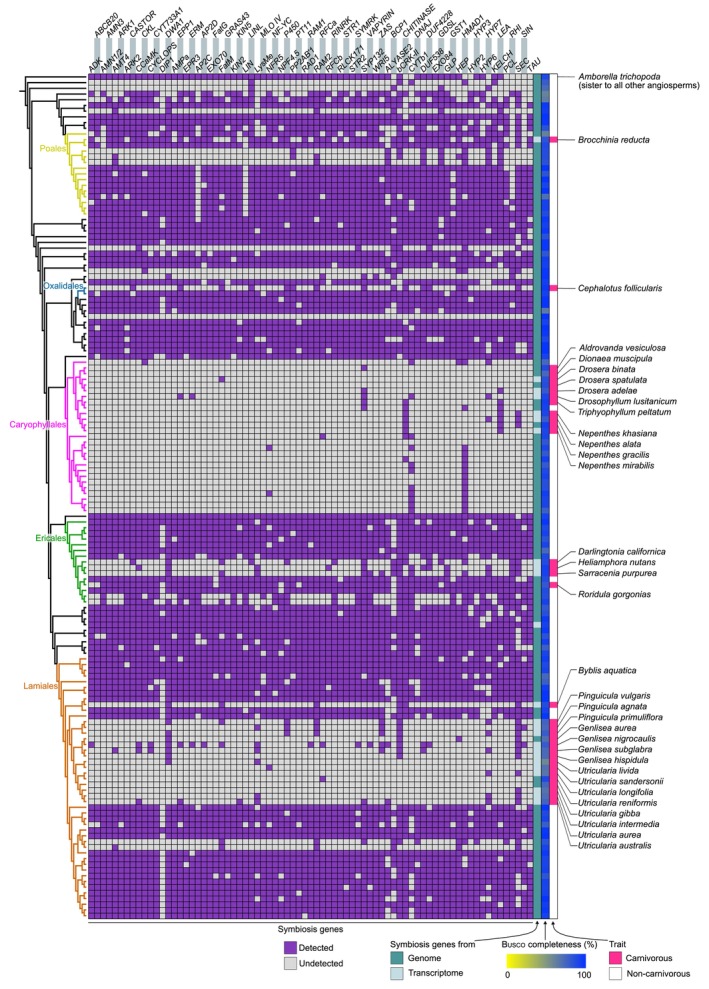
Occurrence of symbiosis genes in carnivorous plants. Symbiosis genes detected across angiosperms are shown. Occurrence reflects the consensus between orthogroups and Blast‐based analyses. Transcriptomes of noncarnivorous plant species are excluded here. For the complete dataset, see Supporting Information Fig. S1.

Altogether, we compiled data from species belonging to 46 out of the 64 recognized angiosperm orders (The Angiosperm Phylogeny Group *et al*., 2016), a substantial increase compared to previous phylogenomic studies on AM symbiosis, which reached 18–20 angiosperm orders (Bravo *et al*., 2016; Radhakrishnan *et al*., 2020). Focusing on the orders where carnivory evolved, we found that most symbiosis genes were undetected in the majority of carnivorous plants, suggesting convergent gene losses (Figs 1, S1). In the Lamiales, symbiosis genes were mostly undetected in all four examined carnivorous genera in the Lentibulariaceae and the Byblidaceae, while they tended to be preserved in the other lineages, including the most closely related non‐carnivorous species in the dataset. A similar situation occurred in the Oxalidales; the carnivorous *C. follicularis* (Cephalotaceae) lacked 80% (60/75) of the symbiosis genes, while starfruit (*A. carambola*, Oxalidaceae), the phylogenetically closest genome in the dataset (estimated divergence time: 66 million years ago (Ma); Saul *et al*., 2023), had a nearly complete repertoire. As such, it appears that the emergence of carnivory coincides with the loss of AM symbiosis in *C. follicularis*, albeit it is difficult to assess which trait change occurred first, as this is the only species in this carnivorous lineage. In the Caryophyllales, all five carnivorous plant genomes lacked the symbiosis genes. In this case, however, we found all the species in the order in our dataset to be devoid of the symbiosis genes. In the Ericales, symbiosis genes were undetected in the transcriptomes of the carnivorous species in the Sarraceniaceae belonging to the genera *Darlingtonia*, *Heliamphora*, and *Sarracenia*. Although transcriptomes generally exhibit lower gene coverage reliability compared to genomes, the symbiosis genes tended to be consistently either detected or undetected across the three genera, suggesting robust gene detection. The symbiosis genes retained in all examined Sarraceniaceae species include, for example, the *SYNTAXIN OF PLANTS132* (*SYP132*) (Huisman *et al*., 2016; Pan *et al*., 2016; Liu *et al*., 2022) and the cuticular wax‐related gene *DROUGHT‐INDUCED WAX ACCUMULATION 1* (*DWA1*) (Zhu & Xiong, 2013). By contrast, the genome of the flypaper‐type carnivorous plant *R. gorgonias*, from the independently evolved carnivorous family Roridulaceae, surprisingly presented most of the symbiosis genes (67/75). Finally, in the Poales, we found another exception where symbiosis genes were well detected in a carnivorous plant, corresponding to the transcriptome of the pitfall‐type carnivorous plant *B. reducta* (Bromeliaceae). Although transcriptomes are usually less complete than high‐quality genome assemblies in terms of gene set coverage, we found 67% (50/75) of symbiosis genes in this species. These include canonical components of the common symbiosis signaling pathway, such as *SYMBIOSIS RECEPTOR‐LIKE KINASE* (*SYMRK*), *CASTOR*, *CALCIUM AND CALMODULIN‐DEPENDENT PROTEIN KINASE* (*CCaMK*), and *CYCLOPS* (Parniske, 2008), and also signaling‐related genes known to function in arbuscule‐containing cells, such as *ARBUSCULE DEVELOPMENT KINASE 1* (*ADK*) (Guo *et al*., 2022; Shi *et al*., 2022), *ARBUSCULAR RECEPTOR‐LIKE KINASE 1* (*ARK1*) (Roth *et al*., 2018; Irving *et al*., 2022), *CYCLIN‐DEPENDENT KINASE‐LIKE (CKL*) (Ivanov & Harrison, 2024), and *RECEPTOR‐LIKE CYTOPLASMIC KINASE 171* (*RLCK171*) (Leng *et al*., 2023).

Besides carnivorous plants, there were certain plant lineages where symbiosis genes were not detected. In the Poales, sedges and rushes from the genera *Juncus*, *Rhynchospora*, and *Carex* lacked most symbiosis genes but also commonly retained some such as *DOMAIN OF UNKNOWN FUNCTION 538* (*DUF538*). In the Ericales, species in the family Ericaceae, known to engage in an alternative mycorrhizal association called Ericoid mycorrhizal symbiosis (Smith & Read, 2008), had retained *c*. 45% of the symbiosis genes in autotrophic species (42–53%, *n* = 3 species). The transcriptomes of two mycoheterotrophic species in this family, *Monotropa hypopitys* and *Monotropastrum humile*, presented 18% and 21% of the symbiosis genes, respectively. In the Lamiales, parasitic species in the Orobanchaceae lacked most symbiosis genes, albeit retaining a few such as the known AM lipid biosynthesis enzymes *FatM* and *REQUIRED FOR ARBUSCULAR MYCORRHIZA 2* (*RAM2*) (Fig. 1).

To explore the contribution of AM symbiosis loss in the overall convergent gene loss in carnivorous plants, we expanded our focus from targeted analyses of known symbiosis genes to the entire orthogroup dataset, asking which genes are commonly lost in carnivorous plant genomes. To this end, we identified orthogroups that are absent from all the carnivorous plant genomes and transcriptomes in the Oxalidales and Lamiales, yet present in representative noncarnivorous genomes of those orders. We selected these two orders from the five analyzed orders for several reasons. First, there are no available carnivorous genomes in the Poales. Second, the Caryophyllales atypically lacks symbiosis genes in both carnivorous and noncarnivorous lineages. Third, the Ericales includes *R. gorgonias*, which maintains the symbiosis genes despite carnivory. Last, Oxalidales and Lamiales represent independent evolutionary origins of carnivory in the rosids and the asterids, the two major eudicot clades. Out of a total of 183 957 orthogroups, 11 274 were commonly present in the target noncarnivorous species, whereas 65 520 were commonly absent in the target carnivorous species. From these, 85 orthogroups fulfilled the criteria of being present in the noncarnivorous species and absent in the carnivorous species. Approximately, one‐third of these 85 orthogroups were associated with AM symbiosis, including 24 focal symbiosis orthogroups analyzed in this study as well as the AM symbiosis‐related genes *ADK2a* (a paralogue of *ADK1*) (Guo *et al*., 2022), *AMMONIUM TRANSPORTER 2;3* (*AMT2;3*) (Breuillin‐Sessoms *et al*., 2015), *MYB1* (Floss *et al*., 2017), and *MYCORRHIZA‐INDUCED GRAS 1* (*MIG1*) (Heck *et al*., 2016) (Fig. S2; Table S3). This result suggests that the loss of symbiosis genes is an important contributor to the convergent and potentially deterministic gene loss in carnivorous plants.

In summary, our dataset lacked the majority of symbiosis genes in 14 carnivorous genera, with the other two genera (i.e. *Brocchinia* and *Roridula*) being exceptions preserving most symbiosis genes. These results suggest that the evolution of plant carnivory tends to accompany the decay of molecular building blocks for AM symbiosis.

### Arbuscular mycorrhizal inoculation assays in carnivorous plants

**Fig. 2:**
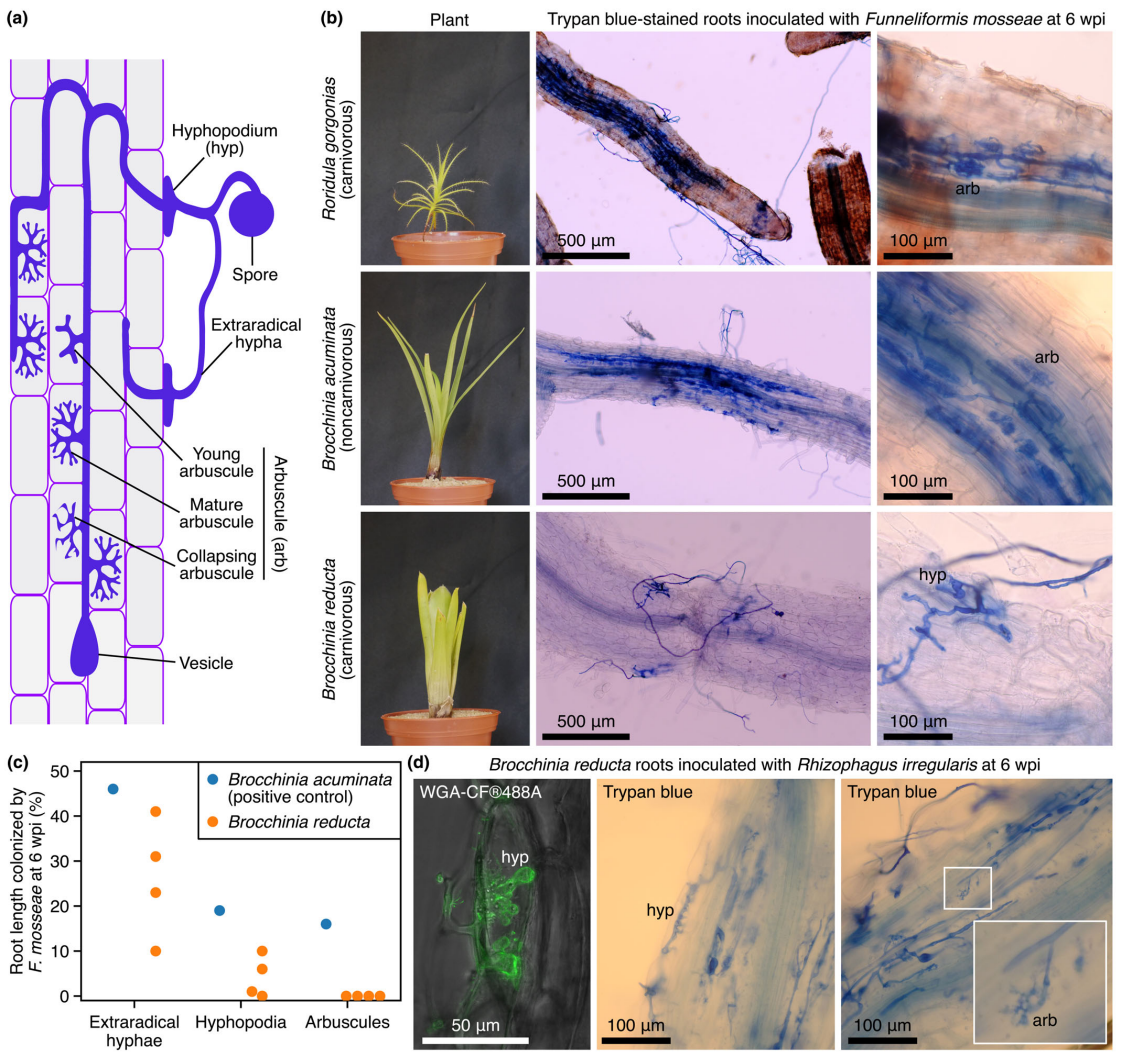
Arbuscular mycorrhizal (AM) inoculation assays in *Roridula* and *Brocchinia*. (a) Schematic depicting AM colonization in a plant root with typical AM fungal structures. (b) Representative images of plants and roots showing *Funneliformis mosseae* colonization. (c) Quantification of AM symbiotic structures. (d) Representative microscopy images of *Rhizophagus irregularis* colonizing *Brocchinia reducta* roots. Supporting Information Fig. S3 displays all additional arbuscules encountered in *B. reducta* roots. wpi, weeks post inoculation.

Given the presence of symbiosis genes in *R. gorgonias* and *B. reducta*, we performed AM inoculation assays to assess the ability of the plants to be colonized by AM fungi and develop arbuscules, the signature symbiotic structures diagnostic for nutrient exchange (Luginbuehl & Oldroyd, 2017; Fig. 2). Plantlets of *R. gorgonias* were inoculated with the AM fungus *F. mosseae*. Despite the difficulty in clearing *R. gorgonias* roots for fungal staining procedures, we successfully observed well‐colonized root zones with arbuscules at 6 wk postinoculation (wpi) (Fig. 2b). This shows that *R. gorgonias* is an AM‐competent species, consistent with its nearly complete repertoire of symbiosis genes.

While *Roridula* comprises exclusively carnivorous species, *Brocchinia* includes both carnivorous and noncarnivorous species. This diversity enables the comparison of AM status between closely related species with and without plant carnivory. To take advantage of this, we examined the carnivorous *B. reducta* and a noncarnivorous relative, *B. acuminata*, for AM colonization ability. *Brocchinia acuminata* exhibited well‐colonized infection sites harboring fully developed arbuscules at 6 wpi. By contrast, several *B. reducta* plants examined showed extraradical hyphae and hyphopodia, but arbuscules were not observed (Fig. 2b,c).

To explore potential differences in colonization ability among fungal species, we performed additional AM inoculation assays in *B. reducta*, employing another AM fungus, *R. irregularis*. The observations were similar to those with *F. mosseae*, but detailed inspections on the rare occurrences of hyphopodia revealed projections into the root epidermis, suggesting difficulties in root entry. In addition, we observed arbuscules, albeit infrequent, small, and stunted (Fig. 2d). The dozen stunted arbuscules encountered in the root systems of three *B. reducta* plants are shown in Fig. S3. In summary, AM competence exists within the genus *Brocchinia*, as evidenced by the noncarnivorous *B. acuminata*. However, the carnivorous *B. reducta* displayed aberrant AM colonization, with infrequent and morphologically altered symbiotic structures. This suggests that *B. reducta* can interact with and be colonized by AM fungi but is unable to establish a successful symbiosis.

Given the mutually exclusive trends observed between AM symbiosis and carnivory, we further investigated the AM status of Stylidium, a plant exhibiting characteristics similar to carnivorous species. *Stylidium*, belonging to the Asterales, was previously proposed to be protocarnivorous based on proteinase activity in its sticky glandular hairs (Darnowski *et al*., 2006). Recent isotope analyses indicated that *Stylidium* species are unlikely to rely on captured organisms for nutrients (Nge & Lambers, 2018). The transcriptome of *S. debile* harbored most symbiosis genes (Fig. S1). In line with this, inoculation assays with *R. irregularis* showed good colonization levels and the formation of numerous arbuscules, supporting the idea of *S. debile* being a noncarnivorous AM‐competent species (Fig. S4).

To confirm the non‐AM status of carnivorous plants lacking a majority of symbiosis genes, inoculation assays were performed in the carnivorous species *C. follicularis*, which resulted in no observed symbiotic structures in its roots (Fig. S5). The retention rates of bioinformatically identified symbiosis genes tightly correlated with experimentally validated AM status, highlighting the effectiveness of using symbiosis gene presence as a reliable predictor of AM competence. Based on this genotype–phenotype association, we infer that other carnivorous lineages with similarly limited symbiosis gene repertoires are also likely to be AM‐incompetent.

### Tissue‐specific expression of retained arbuscular mycorrhizal‐associated genes in *C. follicularis*

**Fig. 3:**
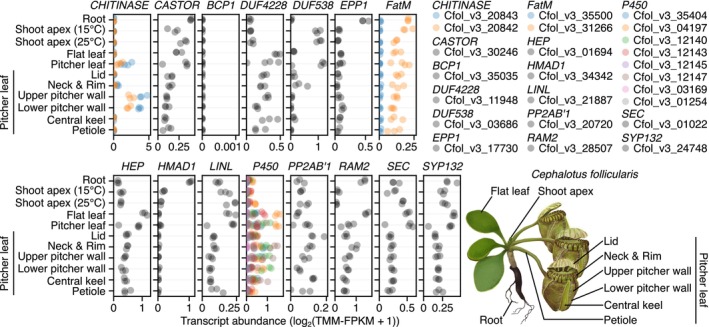
Tissue‐specific expression of retained arbuscular mycorrhizal‐associated genes in the carnivorous pitcher plant *Cephalotus follicularis*. Each point represents a replicate from RNA‐seq experiments. Plant tissues evaluated are listed on the *y*‐axis and are displayed in the accompanying illustration. In the case of shoot apex, RNA was extracted from plants grown at two different temperatures: 15°C and 25°C. Genes are colored gray unless *C. follicularis* possesses multiple orthologs from lineage‐specific duplication, in which case each copy is given a unique color. Illustration by Shun Anzai.

Despite its AM incompetence, we detected 15 symbiosis genes in the carnivorous pitcher plant *C. follicularis*. To infer their functions, we examined previously published tissue‐specific transcriptome data (Fukushima *et al*., 2017; Saul *et al*., 2023). All but two genes, *BLUE COPPER‐BINDING PROTEIN1* (*BCP1*) and one *CYTOCHROME P450* (*P450*) gene, were expressed in the analyzed tissues including aerial parts (Fig. 3), suggesting their roles in non‐AM‐related functions. The two tandemly duplicated *CHITINASE* orthologs (Cfol_v3_20842 and Cfol_v3_20843) were expressed specifically in the trapping pitcher with the highest expression levels in the upper and lower pitcher walls, where multicellular large glands are located on the inner surface (Juniper *et al*., 1989). The chitinase enzyme encoded by Cfol_v3_20843 has been confirmed to be secreted into the digestive fluid (Fukushima *et al*., 2017), suggesting a co‐option for digestive physiology to degrade arthropod chitin. In addition, two of the eight *P450* orthologs (Cfol_v3_12140 and Cfol_v3_12147) showed pitcher‐preferential expression. They belong to a cytochrome P450 subfamily involved in the regulation of disease resistance and apocarotenoid biosynthesis (Wakabayashi *et al*., 2021; Wang *et al*., 2022). *DUF538* (Cfol_v3_03686) also displayed higher expression levels in pitchers compared to flat leaves. While it is tempting to suggest that symbiosis genes may have been repurposed for carnivory, these particular genes identified via phylogenomics have not been functionally characterized in AM symbiosis. This highlights the need for future experimental studies to assess their roles in AM symbiosis in noncarnivorous plant species and to explore their potential co‐option in carnivorous plants.

## Discussion

Our bioinformatic analysis, spanning multiple origins of carnivory and ecological habits, showed that the AM trait has been lost multiple times in carnivorous plant lineages. An earlier phylogenomic study examined only the genome of a single carnivorous plant, *Utricularia gibba* (Bravo *et al*., 2016). Although *U. gibba* was categorized as non‐AM, ambiguity remains regarding the relationship between AM competence and carnivory. This uncertainty arises not only from the lack of data on other carnivorous lineages but also because *U. gibba* is an aquatic herb that lacks roots, a lifestyle incompatible with AM symbiosis. Therefore, our findings establish the mutually exclusive trend between AM competence and carnivory.

Some families in the Caryophyllales such as the Amaranthaceae and Caryophyllaceae are known to be non‐AM (Lambers & Teste, 2013), and a previous study analyzed two genomes from this order, confirming the absence of symbiosis genes (Bravo *et al*., 2016). However, we did not anticipate the near‐complete absence of symbiosis genes across the entire order of our dataset, including 23 families. Some members in the Caryophyllales have been reported to be AM‐competent (Maherali *et al*., 2016), mainly based on information from compilation lists (Wang & Qiu, 2006; Akhmetzhanova *et al*., 2012). However, these compilation lists have been criticized for having misdiagnosed AM types, which are perniciously recycled in subsequent studies (Brundrett & Tedersoo, 2019). Our review of the literature found no convincing evidence of AM colonization and arbuscule formation in the Caryophyllales. Our findings suggest most, if not all, Caryophyllales are non‐AM. Conceivably, the early loss of AM symbiosis provided the potential for the development of alternative nutrient acquisition strategies to evolve in this order. This may include the evolution of ectomycorrhizal symbiosis in several Caryophyllales families (Tedersoo & Brundrett, 2017) and may also be behind the evolution of carnivory, which reaches its maximum genus diversity in this order (Fleischmann *et al*., 2018).

In the carnivorous Ericales, symbiosis genes were mostly undetected in the species in the Sarraceniaceae and mostly detected in *R. gorgonias*. With experimental evidence of AM colonization, *R. gorgonias* emerged as a remarkable outlier to the non‐AM trend observed in carnivorous plants. Mycorrhizal symbiosis in *Roridula*, a genus with two South African species (POWO, 2024), has been largely unexplored, but one century ago, botanist Rudolf Marloth noted in his Flora of South Africa that *Roridula* roots are ‘surrounded by the loose web of a mycorrhiza’ (Marloth, 1925). The biological significance of the AM competence in *Roridula* can at present only be speculated based on its unique traits compared to other carnivorous species. Although *R. gorgonias* is almost entirely covered in sticky hairs that efficiently trap insects, it lacks digestive enzymes and relies on symbiosis with a Hemipteran insect that consumes and digests the trapped prey to subsequently pass the digested nutrients as droppings to the plant (Anderson & Midgley, 2002). Thus, *Roridula* must share the prey‐derived nutrients with symbiotic insects, and this even more unique nutritional mode among carnivorous plants may have led to the maintenance of AM symbiosis as a supplemental source of nutrients.

A total of 50 symbiosis genes were detected in the transcriptome of the carnivorous bromeliad *B. reducta*. Mycorrhizal symbiosis in the genus *Brocchinia*, which comprises 20 species from South America (POWO, 2024), remains largely unknown. Only *B. reducta* corresponds to a confirmed carnivorous species (Givnish *et al*., 1984). Its closest relative within the genus is *B. hechtioides* (Givnish *et al*., 1997), but carnivory in this species has not been thoroughly studied. The family they belong to, the Bromeliaceae, is highly diversified, and the sequencing of internal transcribed spacer 1 (ITS1) revealed its species can host diverse mycorrhizal fungal assemblages in their roots (Leroy *et al*., 2021). One of the most known terrestrial bromeliads, pineapple (*Ananas comosus*), is capable of hosting AM fungi and forming arbuscules (Moreira *et al*., 2015). In our inoculation assays, we observed fully developed arbuscules in the roots of the noncarnivorous *B. acuminata*, demonstrating that AM symbiosis does occur in the genus. However, while the roots of the carnivorous *B. reducta* were able to interact with AM fungi by forming occasional hyphopodia, intraradical hyphae, and arbuscules, symbiotic structures were malformed. The malformed hyphopodia resembled those described in mutants of symbiosis genes such as *VAPYRIN* in other plant species (Reddy *et al*., 2007; Pumplin *et al*., 2010). The few arbuscules encountered were all small and stunted, resembling collapsing arbuscules. Although collapsing arbuscules are common in AM‐competent species, they usually coexist with young and fully developed arbuscules. Environmental factors such as nutrient availability, substrate, and temperature can quantitatively affect colonization patterns, but the inability of *B. reducta* to form fully developed arbuscules suggests a potential genetic impairment. Thus, *B. reducta* colonization patterns may reflect an incipient loss of symbiosis gene functionality, leading to a diminished ability to establish successful AM symbiosis. The notion of an incipient loss of symbiosis genes is further supported by the mutually exclusive pattern between carnivory and AM symbiosis, as *B. reducta* represents the youngest carnivorous lineage (Fleischmann *et al*., 2018). The genus *Brocchinia* itself is young, having emerged *c*. 1.9 Ma, whereas other carnivorous genera are older, with *Nepenthes*, for example, originating nearly 85 Ma. These findings invite further genomic studies across *Brocchinia* species engaging in different lifestyles.

Overall, our findings indicate that AM symbiosis and carnivory do not tend to co‐occur, possibly because each strategy is costly (carbon allocation to fungal partners and investment in photosynthetically inefficient traps, respectively), even though both enhance mineral nutrient acquisition. However, the temporal relationship between the loss of symbiotic ability and the acquisition of carnivory does not always appear to be the same. The mutually exclusive nature of the two traits may thus be explained in different ways. For instance, in lineages like *Brocchinia*, AM symbiosis appears to have been lost upon the evolution of carnivory, suggesting that carnivory offered a more effective strategy for nutrient acquisition in its habitat. By contrast, in the Caryophyllales, AM symbiosis was lost first, and carnivory evolved later. Consequently, the mechanism driving the transition between these two biotic interactions is entirely different in this group. A remote common ancestor relinquished the AM trait, and because the genes required for symbiosis cannot be easily restored or replaced, the lineages leading to extant Caryophyllales gained a broader opportunity to adopt alternative nutritional strategies. This shift may ultimately have paved the way for the evolution of carnivory. This illustrates that diverse evolutionary paths can lead to the convergence of similar trait combinations.

Our analysis also showed nonrandom patterns of symbiosis gene retention. In the Poales, symbiosis genes were mostly absent from the sedges *Carex littledalei* and *Rhynchospora tenuis* (Cyperaceae) and *Juncus effusus* from the related family Juncaceae. Sedges are known to develop dauciform roots specialized for phosphate scavenging, which are analogous to the cluster roots of the Proteaceae (Shane *et al*., 2006). The symbiosis genes retained in these species, such as *ALGINATE LYASE 2* (*ALYASE2*) and *HYPOTHETICAL PROTEIN 7* (*HYP7*) may be pleiotropic, with additional unknown roles beyond symbiosis. In the Ericaceae, we identified a unique set of retained symbiosis genes that differ from those found in the above‐mentioned monocots. Species in this family are known to develop ericoid mycorrhizal symbiosis, where non‐AM fungi form intracellular hyphal coils in epidermal cells (Smith & Read, 2008; Leopold, 2016). The symbiosis genes retained in *Rhododendron delavayi* and *Vaccinium darrowii* may regulate the ericoid mycorrhizal symbiosis in the Ericaceae, while their orthologs in AM hosts regulate the AM symbiosis. The genes absent in the two Ericaceae species may play specific roles in the AM symbiosis in AM‐host species. An earlier survey of some symbiosis genes in the transcriptome of *Rhododendron fortunei* showed *SYMRK*, *CCaMK*, and *CYCLOPS* to be present (Radhakrishnan *et al*., 2020). Our data confirm the presence of these genes in the Ericaceae and expand the known repertoire of symbiosis genes in this family (e.g. *ARK2*, *CASTOR*, *VAPYRIN*). It would be fascinating if retained symbiotic genes were repurposed for alternative nutritional strategies, much like CHITINASE, which is encoded by a symbiotic gene and is secreted into the digestive fluids of the carnivorous plant *Cephalotus*.

In the Lamiales, symbiosis genes were undetected in the Orobanchaceae parasites *Phtheirospermum japonicum* and *Striga asiatica*. Interestingly, the genes were present in the non‐parasitic *Lindenbergia philippensis*, which belongs to the same family. These findings suggest a loss of the AM trait in parasitic plants as well as in dauciform‐forming and ericoid mycorrhizal plants. However, to confirm a widespread absence of symbiosis genes across these different plant guilds, dedicated analyses of each respective trait are necessary, particularly in light of our discovery that *Roridula* serves as an outlier AM species among typically non‐AM carnivorous plants.

AM symbiosis and carnivory are both evolutionarily successful strategies for nutrient acquisition. Understanding the switch between these strategies not only enhances our knowledge of carnivory and AM symbiosis but also elucidates how plants fine‐tune nutrient acquisition for maximal fitness.

## Competing interests

None declared.

## Author contributions

HM and KF conceived the study. HM and MF performed the experiments. HM and KF wrote the manuscript. HM, MF and KF analyzed the data.

## Disclaimer

The New Phytologist Foundation remains neutral with regard to jurisdictional claims in maps and in any institutional affiliations.

## Supporting information


**Fig. S1** Detail of Fig. 1, displaying all species analyzed in this study.
**Fig. S2** Eighty‐five orthogroups absent in the genomes and transcriptomes of carnivorous plant species and present in the noncarnivorous species.
**Fig. S3** *Brocchinia reducta* roots inoculated with *Rhizophagus irregularis* at six wpi stained with Trypan blue.
**Fig. S4** *Stylidium debile* roots inoculated with *Rhizophagus irregularis* at six wpi stained with Trypan blue.
**Fig. S5** *Cephalotus follicularis* roots inoculated with *Rhizophagus irregularis* at six wpi stained with Trypan blue.

**Table S1** Sources and Busco scores of genomes and transcriptomes.
**Table S2** List of symbiosis genes, their associated orthogroup IDs, and gene IDs of the representative species *Medicago truncatula* and *Oryza sativa*.
**Table S3** List of 85 orthogroups absent in carnivorous species and present in noncarnivorous species in the Oxalidales and Lamiales.
Please note: Wiley is not responsible for the content or functionality of any Supporting Information supplied by the authors. Any queries (other than missing material) should be directed to the *New Phytologist* Central Office.

## Data Availability

All data that can be used to replicate the study are available in Tables S1–S3 or on Figshare (doi: 10.6084/m9.figshare.23553813).
