# Supplementary material for: Convergent losses of arbuscular mycorrhizal symbiosis in carnivorous plants
Source: New Phytol. 2025 Sep 25;248(4):2040–51. doi: 10.1111/nph.70544 (PMC12529035; doi:10.1111/nph.70544)
Supplement: Supplementary file 1 — Fig. S1 Detail of Fig. 1, displaying all species analyzed in this study. Fig. S2 Eighty‐five orthogroups absent in the genomes and transcriptomes of carnivorous plant species and present in the noncarnivorous species. Fig. S3 Brocchinia reducta roots inoculated with Rhizophagus irregularis at six wpi stained with Trypan blue. Fig. S4 Stylidium debile roots inoculated with Rhizophagus irregularis at six wpi stained with Trypan blue. Fig. S5 Cephalotus follicularis roots inoculated with Rhizophagus irregularis at six wpi stained with Trypan blue. [file NPH-248-2040-s001.pdf]

***New Phytologist* Supporting Information**

Article title: **Convergent losses of arbuscular mycorrhizal symbiosis in carnivorous plants**
Authors: Héctor Montero, Matthias Freund, Kenji Fukushima
Article acceptance date: 9 August 2025

The following Supporting Information is available for this article:

Supplementary Tables 1–3 (separate file)
Supplementary Figs. 1–5

Supplementary Tables

Supplementary Table 1. Sources and BUSCO scores of genomes and transcriptomes employed in our analysis. For the procedure to obtain BUSCO scores, see “Species tree inference” in the Methods section.

Supplementary Table 2. **List of symbiosis genes, their associated orthogroup IDs, and gene IDs of the representative species *Medicago truncatula* and *Oryza sativa*.** For the procedure to create the list of symbiosis genes, see “Symbiosis gene set” in the Methods section.

Supplementary Table 3. List of 85 orthogroups absent in carnivorous species and present in non-carnivorous species in the Oxalidales and Lamiales. For the procedure to obtain the different orthogroups, see “Orthogroup tree inference” in the Methods section of the main article text. This file lists orthogroups that were absent in all the carnivorous plant genomes and transcriptomes in the Oxalidales and Lamiales (corresponding to *Cephalotus follicularis*, and all species in the genera *Byblis*, *Genlisea*, *Pinguicula* and *Utricularia*), and present in the representative non-carnivorous genomes in the Oxalidales and Lamiales (corresponding to *Averrhoa carambola*, *Andrographis paniculata* and *Erythranthe guttata*).
Columns:

- Gene name: Names of AM genes as used throughout this manuscript
- Orthogroup: Orthogroup ID assigned by SonicParanoid
- UniProt best hit: Annotation from the UniProt BLAST best hit for a representative gene in each orthogroup
- Abronia_nealleyi to Zea_mays: Comma-separated gene IDs for each species

Supplementary Figures

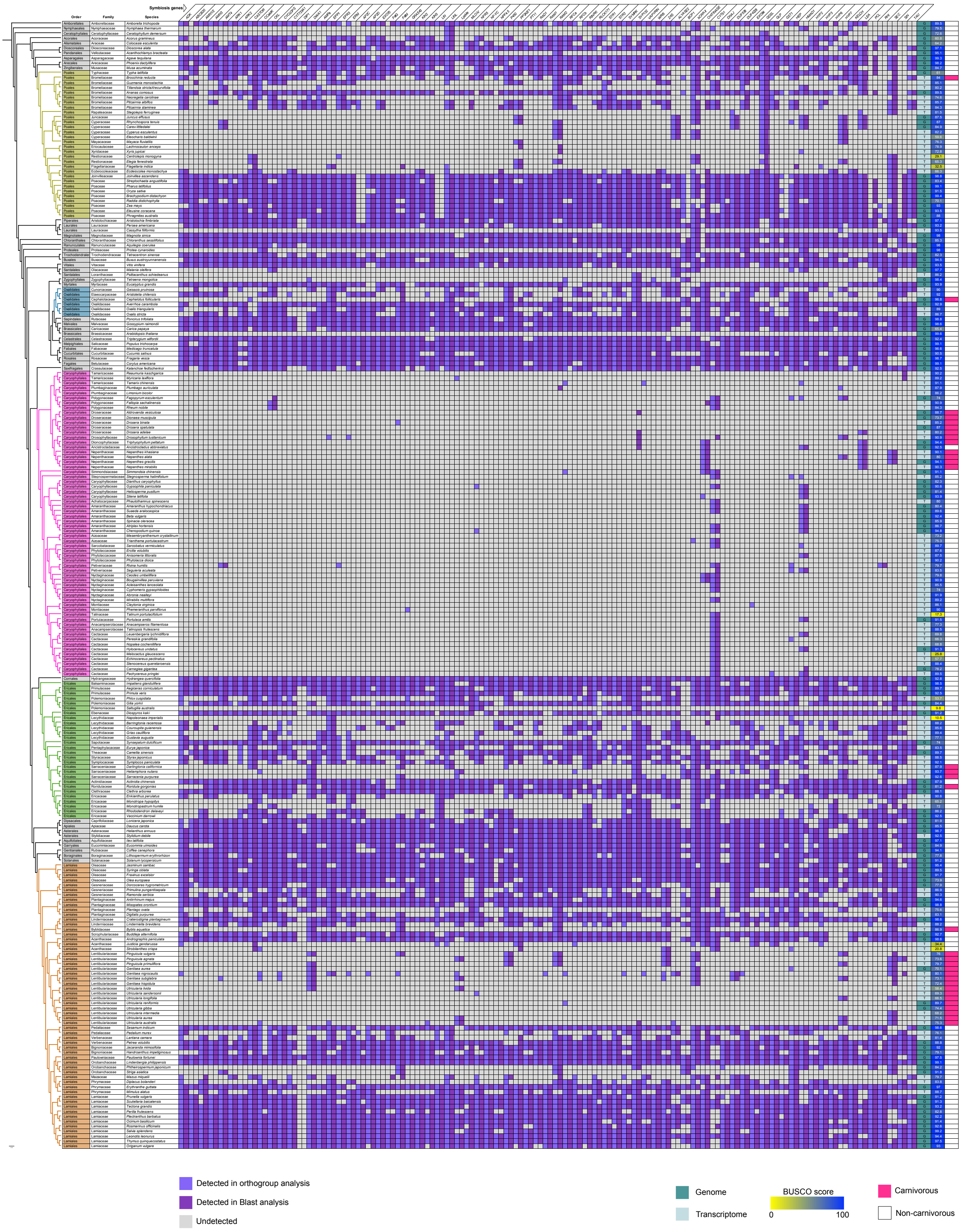

Supplementary Figure 1. Detail of Figure 1, displaying all species analysed in this study.

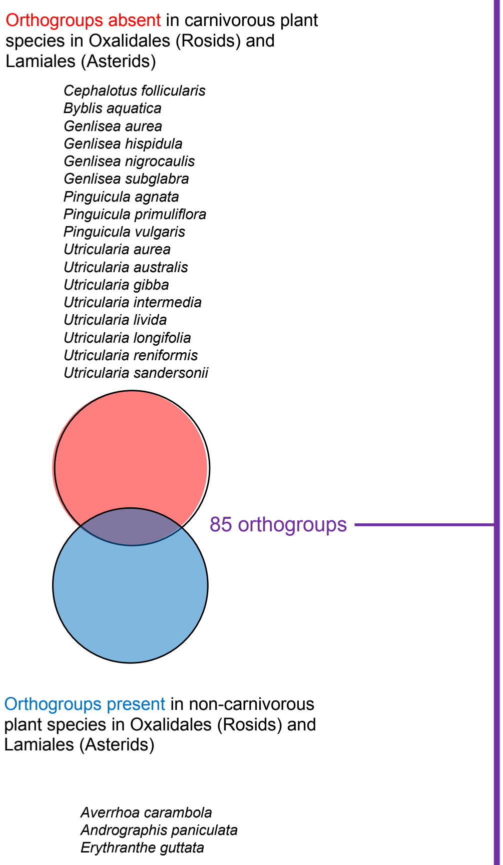

Gene name	Orthogroup
	SP0000531
	SP0001051
PCL	SP0001115
MIG1	SP0001356
	SP0002897
	SP0003896
RHI	SP0004249
	SP0005197
	SP0005241
WRI5	SP0005961
ARK1/ARK2	SP0006504
	SP0007204
	SP0007342
STR1/STR2	SP0008019
	SP0008346
	SP0008767
	SP0008854
GDSL	SP0009257
	SP0010824
	SP0010929
	SP0011028
CKL	SP0011256
RINRK	SP0011515
	SP0011623
	SP0012048
	SP0012792
	SP0013005
	SP0013056
	SP0013122
	SP0013369
	SP0013956
	SP0014020
EXO84	SP0014071
HYP3	SP0014074
	SP0014229
	SP0014366
	SP0014562
PT11	SP0014690
KELCH	SP0014694
RFCb	SP0014749
	SP0014996
EPR3	SP0015046
	SP0015509
CYTb1	SP0015561
	SP0015577
	SP0015656
RLCK171	SP0015814
	SP0016891
	SP0016953
NF-YC	SP0017106
ADK2a	SP0017286
CYT733A1	SP0017302
KIN2	SP0017580
MYB1	SP0017649
FatG	SP0017794
EXO70	SP0017958
PP2AB'1	SP0018048
AMPa	SP0018053
	SP0018126
GRAS43	SP0018218
	SP0018266
	SP0018384
	SP0018388
	SP0018674
	SP0019159
	SP0019285
	SP0019300
	SP0019598
	SP0019683
	SP0019785
	SP0020115
	SP0020452
	SP0020547
	SP0020764
	SP0021034
	SP0021396
	SP0021622
	SP0021641
	SP0021680
	SP0021812
AMT2;3	SP0022363
	SP0022731
	SP0024801
	SP0025116
	SP0039109

Supplementary Figure 2. 85 orthogroups absent in the genomes and transcriptomes of the carnivorous plant species *Cephalotus follicularis*, *Byblis aquatica*, *Genlisea aurea, G. hispidula, G. nigrocaulis, G. subglabra, Pinguicula agnata, P. primuliflora, P. vulgaris*, *Utricularia aurea*, *U. australis*, *U. gibba*, *U. intermedia*, *U. livida*, *U. longifolia*, *U. reniformis* and *U. sandersonii;* and present in the non-carnivorous genomes of *Averrhoa carambola*, *Andrographis paniculata* and *Erythranthe guttata*. Of these, 28 orthogroups correspond to AM-related genes (named next to the orthogroup IDs). Table displaying gene IDs per each species can be found in Supplementary Table 2.

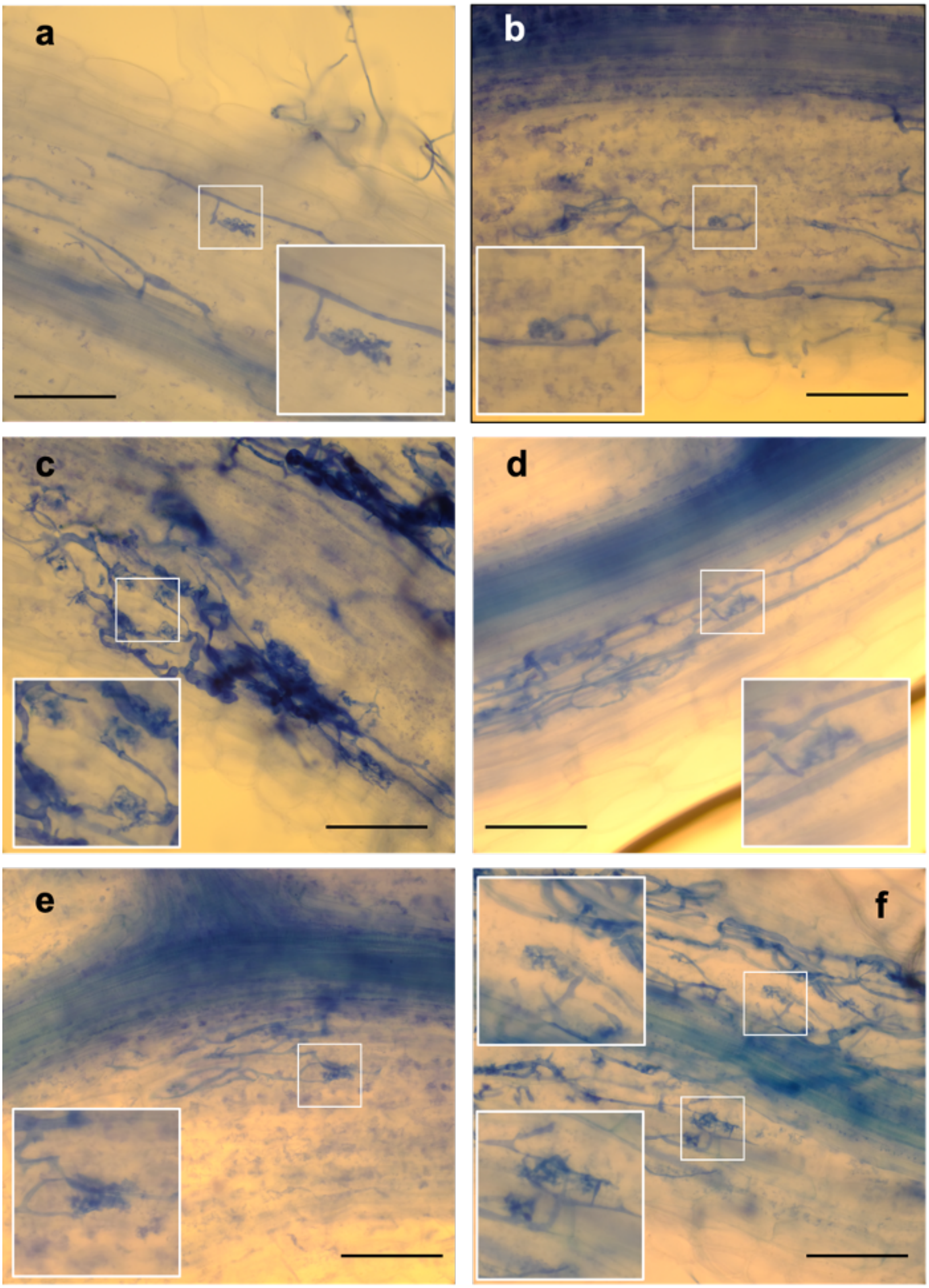

Supplementary Figure 3. *Brocchinia reducta* roots inoculated with *R. irregularis* at six wpi stained with Trypan blue. All root areas colonized and forming arbuscules along the root system of three plants scored are shown. Arbuscules are boxed and images enlarged in insets. **a**, **b**, **d** and **e** show single stunted arbuscules in sparsely colonized root zones. **c** and **f** show multiple stunted arbuscules in root zones with more intraradical hyphae. Scale bar, 100 µm.

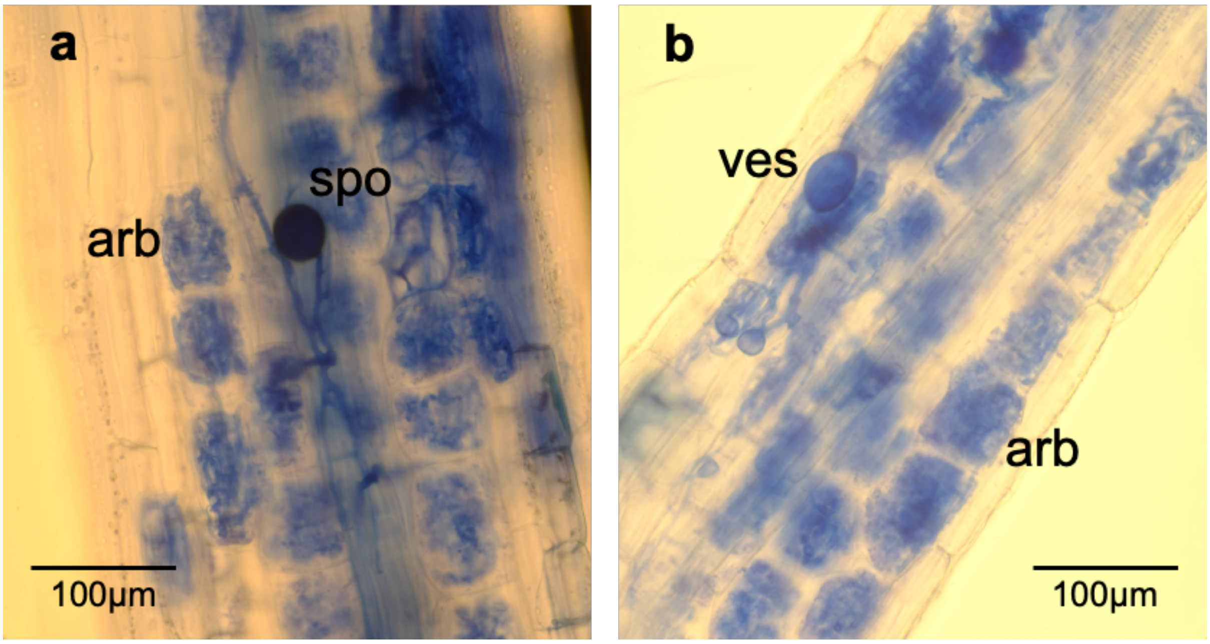

Supplementary Figure 4. *Stylidium debile* roots inoculated with *R. irregularis* at six wpi stained with Trypan blue. **a**, arbuscules (arb) and spore (spo). **b**, arbuscules and vesicle (ves).

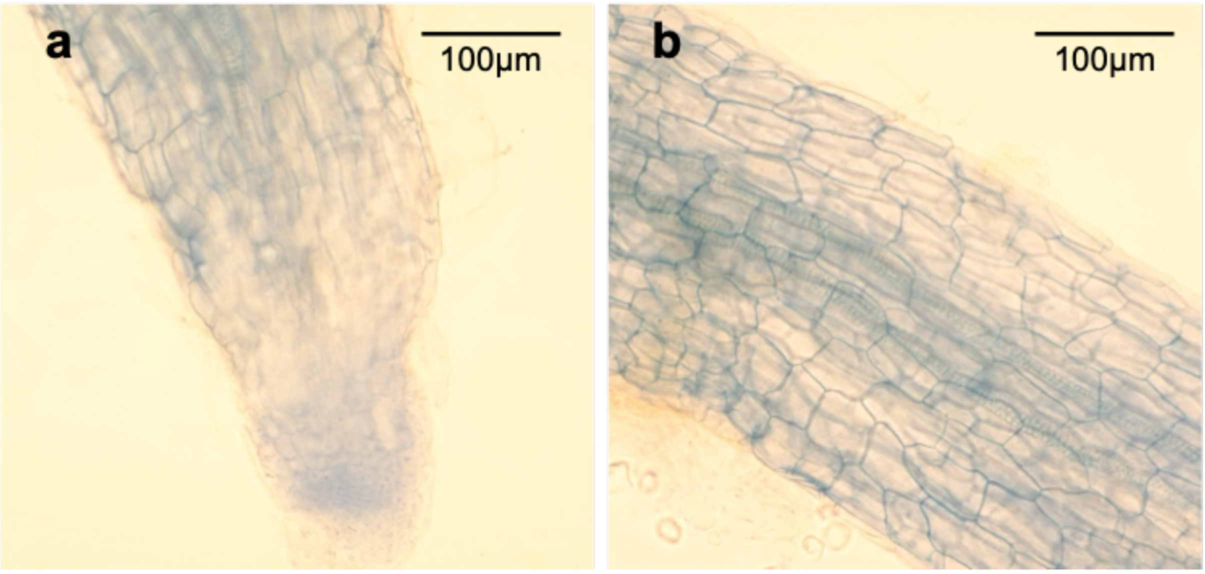

Supplementary Figure 5. *Cephalotus follicularis* roots inoculated with *R. irregularis* at six wpi stained with Trypan blue. **a**, root tip. **b**, root area distal from tip.
